# Limbic-predominant neuroimaging correlates of plasma p-Tau217 in preclinical and clinical Alzheimer’s disease

**DOI:** 10.3389/fnagi.2026.1748524

**Published:** 2026-04-22

**Authors:** Ankit Patel, William Guiler, Sam Pepper, Riley E. Kemna, Paul J. Kueck, Ian W. Weidling, Hana D. Mayfield, Casey S. John, Dinesh Pal Mudaranthakam, Heather M. Wilkins, Russell H. Swerdlow, Jeffrey M. Burns, Jill K. Morris, Robyn A. Honea

**Affiliations:** 1 University of Kansas Alzheimer’s Disease Research Center, University of Kansas Medical Center, Kansas City, KS, United States; 2 Department of Neurology, University of Kansas School of Medicine, Kansas City, KS, United States; 3 Department of Biostatistics and Data Science, University of Kansas Medical Center, Kansas City, KS, United States; 4 Department of Biochemistry and Molecular Biology, University of Kansas Medical Center, Kansas City, KS, United States

**Keywords:** centiloid, cortical thickness, FDG-PET, hippocampus, p-tau217, voxel-based morphometry

## Abstract

**Background:**

Plasma phosphorylated tau at threonine 217 (p-Tau217) has emerged as a highly sensitive and specific blood-based biomarker for Alzheimer’s Disease (AD). However, its regional brain correlates with multimodal neuroimaging, beyond tau-PET, remain underexplored, particularly in early disease phases where limbic involvement may predominate. This study aimed to map the associations of plasma p-Tau217 with amyloid-PET burden, FDG-PET metabolism, and structural brain morphometry in a well-characterized cohort spanning cognitively unimpaired (CU) and cognitively impaired (CI) individuals across AD dementia stages, hypothesizing increased AD neuroimaging biomarkers in individuals with elevated p-Tau217.

**Methods:**

We analyzed data from 259 participants from the University of Kansas Alzheimer’s Disease Research Center (KU ADRC) Clinical Cohort. Imaging outcomes included amyloid-PET, FDG-PET, gray matter volumetric regions of interest as well as whole brain voxel-based morphometry (VBM), and surface-based morphometry (SBM) for cortical thickness (CT), sulcal depth (SD), gyrification index (GI), fractal dimension (FD). Analyses were stratified by diagnostic and pTau-217 positivity (CU pTau + , CU pTau -, CI pTau + and CI pTau-). Spearman’s correlations and voxel/surface-wise regressions evaluated p-Tau217 associations with imaging metrics, accounting for age and sex.

**Results:**

Plasma p-Tau217 was elevated in CI versus CU. Individuals with elevated plasma p-Tau217 had increased amyloid-PET deposition across the cortex, as well as significantly higher centiloids, both in CU and CI individuals. In CI individuals, elevated p-Tau217 was associated with reduced voxel-wise gray matter volume and cortical thickness in limbic, temporal, parietal, and frontal regions, plus increased cingulate FD. In both CU and CI pTau + individuals, p-Tau217 correlated with AD Signature gray matter decreases.

**Conclusion:**

These findings show that CU and CI individuals with elevated plasma p-Tau217 have both increased amyloid-PET burden but also temporolimbic gray matter atrophy and hypometabolism. This supports p-Tau217 as a minimally invasive, scalable biomarker for early AD detection, risk stratification, and prognostic monitoring in preclinical stages, potentially guiding trial enrichment and personalized interventions.

## 1 Introduction

Alzheimer’s disease (AD) is a prolonged biological process that begins well before clinical symptoms. Pathology accumulates during a window when cognition can appear stable, and clinical practice guidelines rely heavily on positron emission tomography (PET) or cerebrospinal fluid (CSF) sampling, which are expensive or invasive for broad use. Scalable blood tests are, therefore, attractive for early detection and triage. Plasma-based markers of phosphorylated tau (p-tau) are elevated in early-AD and can discriminate between AD and other neurodegenerative diseases (Palmqvist et al., 2020; Leuzy et al., 2022). Among available blood biomarkers, phosphorylated tau at threonine 217 (p-Tau217) has shown consistent performance across platforms and settings and has been reported to be elevated from preclinical to clinical disease stages (Thijssen et al., 2021). P-Tau217 shows high diagnostic accuracy and robust agreement with tau-PET across platforms and laboratories (Ashton et al., 2024; Groot et al., 2024; Mendes et al., 2024). Moreover, studies in both clinical and population cohorts demonstrate that higher p-Tau217 aligns with amyloid positivity and predicts near-term cognitive decline, including among CU adults (Janelidze et al., 2024; Palmqvist et al., 2024; Ossenkoppele et al., 2025). A systematic review indicates that p-Tau217 meets or exceeds other plasma phosphorylated tau species for identifying amyloid and tau status across the AD continuum (Antonioni et al., 2025). Given that a majority of individuals first receive FDG-PET, Amyloid-PET, and/or structural MRI during a clinical visit, it is essential to understand how plasma p-Tau217 levels relate to various multimodal neuroimaging signatures beyond tau-PET, particularly in the earliest stages and throughout disease progression.

A blood measure can index disease activity, but to stage disease and to guide imaging interpretation, we also need to understand where biology expresses first in the brain. Structural neuroimaging, amyloid-PET, and fluorodeoxyglucose (^18^F) positron emission tomography (FDG-PET) in tandem can provide a coherent map of early involvement. P-Tau217 is positively associated with amyloid burden measured on PET in cognitively unimpaired (CU) individuals (Cogswell et al., 2025), individuals with AD (Janelidze et al., 2025; Shin et al., 2025) and has been correlated with amyloid PET-based stages of disease progression (Montoliu-Gaya et al., 2025). Across diverse cohorts, the earliest and most reliable relationships of plasma measures of plasma tau to amyloid-PET and tau-PET appear in the medial temporal lobe (MTL), including the entorhinal and hippocampal formation, before involvement extends into lateral temporal cortex and posteromedial association regions (La Joie et al., 2020; Sanchez et al., 2021; Groot et al., 2023). FDG-PET measures cortical hypometabolism, with hypometabolism in the posterior cingulate, precuneus, and in the lateral temporoparietal cortex often indicating AD pathology (Quispialaya et al., 2025), and those reductions broaden with advancing stage (Jagust, 2018). Studies of FDG-PET and p-Tau217 have shown a relationship between global hypometabolism and plasma p-Tau217; however, this relationship has not been examined regionally (Wang et al., 2025).

Thus, it is key to localize the regionally specific relationships of p-Tau217 to early changes in brain AD biomarkers, even before cognitive decline. Cross-sectional studies in AD dementia show that cortical thinning in temporal and posteromedial regions relates to both tau and amyloid burden, and that combined models of pathology and structure explain more variance in cognition than either modality alone (Bejanin et al., 2017; Ossenkoppele et al., 2019; Sun et al., 2019). Longitudinally, selective increase of p-Tau217 over other plasma markers (ptau231, amyloidb42/40 ratio) has been associated with cognitive decline and atrophy in preclinical AD in multiple cohorts (Ashton et al., 2022). P-Tau217 positivity has been associated with reduced cortical thickness regardless of cognitive symptoms in older adults (Brickman et al., 2022). This study aimed to measure whether there were increased AD neuroimaging biomarkers in individuals with elevated p-Tau217, in a well-characterized cohort comprising cognitively unimpaired (CU) and cognitively impaired (CI) individuals across AD dementia stages, hypothesizing that individuals with elevated p-Tau217 exhibit increased AD neuroimaging biomarkers. We also sought to multimodally map how plasma p-Tau217 relates to structural, amyloid-PET, and FDG-PET across the clinical continuum. Most neuroimaging studies have focused on *a priori* brain regions, especially with brain structure; thus, we exploit rich dimensionality by analyzing voxel-based and surface-based regional relationships with p-Tau217, and we analyze regions of interest (ROIs) alongside whole brain maps. We hypothesized subtle but detectable limbic associations across structural, amyloid-PET and FDG-PET imaging in CU adults, with clearer temporolimbic patterns in AD dementia (Ferreira et al., 2020).

## 2 Materials and methods

### 2.1 Standard protocol approvals, registrations, and patient consents

Study procedures were approved by the University of Kansas School of Medicine Institutional Review Board and were in accordance with U.S. federal regulations. All participants provided written informed consent.

### 2.2 Participants

Participants were recruited for the intervention and observational studies at the University of Kansas Alzheimer’s Disease Research Center (KU ADRC) and were part of the Clinical Cohort, which included over 490 individuals. The cohort includes participants with cognitive impairment and healthy cognition. CU individuals were included at age 60 and older. The Uniform Data Set (UDS) is administered to ADC Clinical Cohort participants approximately annually. Individuals were included in this retrospective analysis if they underwent brain imaging and had blood p-Tau217 analysis as part of these ongoing observational studies on aging and risk for AD (ClinicalTrials.gov: NCT01129115, NCT02000583, NCT00267124). A total of 259 individuals had MRI and plasma data; subsets included 51 with Amyloid-PET and 67 with FDG-PET, all with blood collection within 6 months of imaging.

All participants also underwent a standard examination, which included a thorough clinical and cognitive evaluation with a clinician at the KU ADRC. This clinical evaluation consists of a semi-structured interview (Clinical Dementia Rating, CDR) with the participant and study partner (Morris, 1993) and a physical and neurological examination. Clinical evaluation results were used to verify CU status, which were reviewed along with psychometric battery results and finalized at a consensus diagnostic conference attended by clinicians and psychometricians using the NINCDS-ADRDA criteria as well as the McKann NIA-AA workgroup diagnostic guidelines (McKhann et al., 2011; Beach et al., 2012). Individuals were excluded from participating if they had other neurological disorders that could impair cognition, evidence of bleeding disorders during screening, clinically significant disease, psychiatric disorder, systemic illness, stroke, or myocardial infarction.

A psychometrician administered a standard psychometric battery as described in a previous publication (Weintraub et al., 2009). As published previously, we used Mplus to combine test scores into cognitive domain-specific factor scores using confirmatory factor analysis, and specific tests were organized by whether they measured attention, verbal memory, or executive function (Watts et al., 2019). Domain-specific factor scores were used as descriptive variables in our demographics analysis. Other covariates included the Geriatric Depression Scale (GDS), the Montreal Cognitive Assessment (MoCA), and the Mini-Mental State Examination (MMSE).

### 2.3 Genotyping and plasma marker procedures

Determination of Apolipoprotein E (*APOE)* genotype was performed by the National Cell Repository for Alzheimer’s Disease (NCRAD), with independent verification of selected samples by the KU ADRC Biomarker Core using a previously described allelic discrimination assay (Wilkins et al., 2017). APOE genotypes were determined by SNP analysis (rs429358 and rs7412). The presence of one ε4 allele determined APOE ε4 positivity.

Additional blood was collected using EDTA as an anticoagulant and centrifuged at 1,800 × g to generate plasma. Samples were frozen at −80C before analyses. pTau-217 was measured using the AlzPath—assay using a Simoa HD-X (Quanterix, Billerica, MA) according to manufacturer directions, with appropriate quality control samples. All samples were run in duplicate, and the mean concentration of the blood biomarkers was recorded from each blood sample. Samples with pTau-217 values > 0.63 pg/mL were considered to be “positive” as previously shown using a three-range reference for Aβ positivity (Ashton et al., 2024). We grouped individuals within the CI and CU diagnostic groups as pTau-217 + (pTau + , > = 0.63) and pTau-217- (pTau-, < 0.62)

### 2.4 Amyloid PET

PET scans were obtained approximately 50 min after administration of intravenous florbetapir 18F-AV45 (370 MBq) or florbetaben 18F tracers on a GE Discovery ST-16 PET/CT scanner. Data were acquired as a dynamic 90-min series (6 × 5-min plus 6 × 10-min frames) reconstructed to 2mm isotropic resolution. Two PET brain frames of 5 min in duration were acquired continuously, summed, and attenuation corrected. Frames from 50 min post-injection were averaged to generate static images, which were co-registered to the participant’s T1-weighted MRI using custom processing using the Computational Anatomical Toolbox 12 (CAT12 Version 12.6, C. Gaser, Structural Brain Mapping Group, Jena University Hospital, Jena, Germany; ^1^ and Statistical Parametric Mapping version 12 (SPM12; Wellcome Trust Centre for Neuroimaging, London, United Kingdom; ^2^ that operate under Matlab (R2023b) (the Mathworks, Natick, MA) and smoothed with a 6mm full-width at half-maximum (FWHM) Gaussian kernel. Standardized uptake value ratios (SUVRs) were calculated for composite regions encompassing the frontal, posterior cingulate, temporal, and lateral parietal cortices relative to a whole-cerebellum reference. Centiloid scaling was performed via tracer-specific linear transformations, consistent with the Centiloid Project protocol. Final Centiloid values and composite regional SUVRs were used for statistical analysis. We had 51 individuals who underwent Amyloid-PET scans and also participated in the blood plasma collection protocol, had blood collection within 6 months of their Amyloid-PET, and passed quality control.

### 2.5 FDG-PET acquisition and processing

FDG-PET scans were acquired on a GE Discovery ST-16 PET/CT scanner. A low-dose CT transmission scan was performed before PET scanning. Then 2 × 4 min frames were acquired, reconstructed, and attenuation corrected to a single FDG-PET image. Processing included rigid-body co-registration to the corresponding T1-weighted MRI using SPM12 and CAT12 to create segmented, individual gray matter regions of interest, followed by spatial smoothing with a 6 mm FWHM Gaussian kernel. SUVRs were calculated using a composite reference region comprising the pons and cerebellar vermis. Regional SUVRs were extracted for the whole gray matter, the anterior cingulate cortex, precuneus, primary motor, superior medial frontal cortex, superior parietal cortex, posterior cingulate cortex, parahippocampus, hippocampus, amygdala, cerebellum, pons, and middle frontal cortex using the Neuromorphometrics atlas to define regions of interest^3^ (Tzourio-Mazoyer et al., 2002). Quality control criteria included exclusion of scans with motion artifacts or incomplete scans. We had 67 individuals with FDG-PET scans and who also participated in the blood plasma collection protocol, had blood collection within 6 months of their FDG-PET, and passed quality control.

### 2.6 MRI

Magnetic resonance imaging (MRI) was done on a Siemens Skyra scanner. We obtained a high-resolution T1-weighted image (MPRAGE; 1 × 1 × 1 mm voxels; TR = 2,500 ms, TE = 4.38 ms, TI = 1,100, FOV = 256 × 256 with 18% oversample, 1mm slice thickness, flip angle 8deg) for detailed anatomy with high gray-white matter contrast. Every scan was checked for image artifacts and gross anatomical abnormalities. This study included 259 individuals with MPRAGE scans and who also participated in the blood plasma collection protocol, had blood collection within 6 months of their MRI, and passed quality control.

For VBM and SBM analysis and pre-processing of T1-weighted images, we used the Cat12 and SPM12, which operate under Matlab (R2023.b) (The Mathworks, Natick, MA) on Mac. This was used for brain volume (VBM) and surface-based measures such as cortical thickness (CT), sulcal density (SD), GI (gyrification index), and fractal dimension (FD). All the SBM procedures^4^ were conducted using default settings.

T1 images were corrected for bias-field inhomogeneities, registered using linear (12-parameter affine) and non-linear transformations, spatially normalized using the high-dimensional DARTEL algorithm into MNI space (Ashburner, 2007), and segmented into gray matter (GMV), white matter (WMV), cerebrospinal fluid (CSF), and white matter hyperintensity (WMH). We calculated total intracranial volume (TIV) using gray, white, and CSF volumes. Gray matter regions of interest were extracted using the Neuromorphometrics atlas. GMV, WMV, WMH, HOC, and AD Signature were used as key metrics for statistical analyses. HOC is the mean of the LHOC (left hippocampal gray matter divided by the sum of left hippocampal gray matter and left inferior lateral ventricles) and RHOC (right hippocampal gray matter divided by the sum of right hippocampal gray matter and right inferior lateral ventricles). We also calculated an AD Signature composite, which included gray matter medial (hippocampus, parahippocampus, entorhinal cortices), middle, and inferior and superior temporal cortex volumes (Jack et al., 2017; Moscoso et al., 2021).

### 2.7 Statistical analyses

Statistics were performed using IBM SPSS Statistics version 29.0.0.0, RStudio (2025.05.01). Descriptive statistics were calculated for each of the 4 groups; CU pTau + , CU pTau-, CI pTau + and CI pTau-. Group assignments were based on plasma p-Tau217 elevated and non-elevated cutoffs described above, and clinical diagnosis, with CI individuals including both MCI and AD diagnoses. Continuous variables were summarized using means and standard deviation, and between-group differences were evaluated using the analysis of variance (ANOVA) statistical test. Categorical variables were summarized into counts. Between-group differences were evaluated using the chi-square statistical test. Univariate analyses were conducted to test for between CU/CI Ptau ± group differences in neuroimaging metrics across Amyloid-PET lobe-based SUVR and Centiloid values, FDG-PET regional SUVR values, and T1-derived metrics of AD Signature GM volume, WMH, HOC, GMV, and WMV, with all analyses including age and sex as covariates. Bonferroni-corrected *post-hoc* tests for specific group comparisons were also completed (CU pTau- vs. CUpTau + ; CU pTau- vs. CI pTau-; CU pTau- vs. CI pTau + ; CU pTau + vs. CI pTau-; CU pTau + vs. CI pTau + ; CI pTau- vs. CI pTau +). Spearman correlation analyses (two-tailed) were conducted to examine the regional relationships between plasma p-Tau217 and Amyloid-PET lobe-based SUVR and Centiloid values, FDG-PET regional SUVR values, and T1-derived metrics within CU/CI pTau + /pTau- groups. *P*-values were corrected for a false discovery rate using the Benjamini and Hochberg method.

For VBM and SBM analyses, the modulated gray matter segmentations were smoothed using a 12 mm FWHM before group-level voxel-wise and vertex-wise analysis. Resampled surface data for cortical thickness (CT) were smooth to 15 mm FWHM kernel, and fractal dimension (FD), and sulcal depth (SD, and data for gyrification were smoothed using a 25 mm FWHM kernel prior to 2*^nd^* level analyses. For all analyses, voxels are reported in the MNI standard space in SPM12. We used a multiple regression model to investigate associations between p-Tau217 and gray matter volume, surface-based CT, FD, SD, and GI, included age, sex, and total intracranial volume (TIV, VBM only) as covariates of no interest. Statistics were done in imaging space across all voxels. We did analyses across all diagnostic groups, and within CU, CU pTau + , CI, and CI pTau + groups. Family-wise error (FWE) correction was applied across the entire brain, and we considered *p* < 0.05 after adjustment as significant.

## 3 Results

From a total of 259 participants, 138 participants were CU (CU pTau + = 36), 121 were considered CI (CI pTau + = 82), there were significant differences in age and sex across groups, with the CU pTau - group having a higher proportion of females (*n* = 71) than males (*n* = 31) compared to the other 3 groups. Plasma p-Tau217 had a mean of 0.338 ± 0.13 for CU pTau-, 1.034 ± 0.38 for CU pTau + , 0.395 ± 0.13 for CI pTau– and 1.162 ± 0.53 for CI pTau + , with a significant difference across groups (*F* = 98.517, *p* < 0.001). There were also significantly different proportions of APOE ε4 carriers with the highest percentage of ε4 in the CU pTau + group. There was no difference in education between diagnosis groups. Demographics are presented in Table 1.

**TABLE 1:** Demographics by diagnosis group.

Variable	CU pTau- (*n* = 102)	CU pTau + (*n* = 36)	CI pTau- (*n* = 39)	CI pTau + (*n* = 82)	*p*-value	Statistic
Age, mean (SD)	72.91 (6.1)	77.78 (7.8)	73.31 (7.5)	75.93 (7.3)	< 0.001	*F* = 5.964
Sex, M/F	31/71	17/19	21/18	48/34	0.001	χ^2^ = 16.156
Education, years	16.62 (2.3)	16.83 (3.0)	16.74 (3.6)	16.22 (2.8)	0.622	*F* = 0.590
APOE ε4, neg/pos	72/30	23/13	21/18	32/46	< 0.001	χ^2^ = 38.813
Plasma p-Tau217	0.338 (0.13)	1.034 (0.38)	0.395 (0.13)	1.162 (0.53)	< 0.001	*F* = 98.517

Demographic, APOE ε4 and plasma p-Tau217 characteristics of the CU p- Tau-, CU p-Tau ^+^ , CI p-Tau -and CI p-Tau + individuals from the VBM and SBM analysis. Values are mean [SD (standard deviation)] unless otherwise indicated. Group comparisons conducted using ANOVA for continuous variables and χ^2^ tests for categorical variables. M, male; F, female.

**FIGURE 1:**
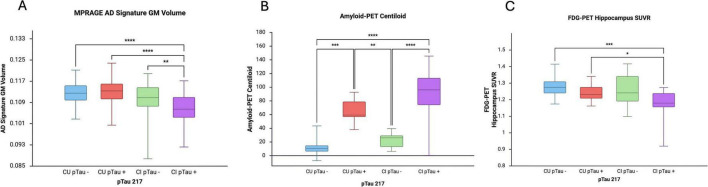
Differences across clinical groups in select metrics, **(A)** gray matter Alzheimer’s Signature regions, **(B)** Amyloid-PET Centiloid, and **(C)** FDG-PET Hippocampal SUVR for groups defined by pTau-217 cutpoint. Full statistical values listed in Table 2 for all imaging metrics from ANOVA run with age and sex as covariates. Significance noted from significant Bonferroni *post-hoc* tests between diagnosis and pTau groups. *P < 0.05, **P < 0.01, ***P < 0.001, ****P < 0.0001.

Univariate analyses were conducted to test for between CU/CI pTau ± group differences in neuroimaging metrics across the three neuroimaging modalities. There was a statistically significant differences between the CU/CI pTau ± in all Amyloid-PET SUVR regions and Centiloid value (Figure 1 and Table 2). *Post-hoc* tests showed that this was driven by increases in amyloid-PET deposition in the CU pTau + and CI pTau + groups (Figure 1 and Table 2). Within the FDG-PET regional SUVR values, there was a significant overall difference between the 4 groups in the precuneus, superior frontal gyrus, posterior cingulate cortex, parahippocampal gyrus, hippocampus, amygdala and middle frontal cortex, with *post-hoc* tests showing that this was particularly driven by the lower glucose metabolism across regions in the CI pTau + group compared to the CU pTau- group. Among the structural MPRAGE metrics, AD Signature, hippocampal occupancy score (HOC) and overall GMV were significantly different between all 4 groups with AD Signature and HOC showing progressive decreased values in the gray matter volumes across diagnosis and pTau groups, with CI pTau + groups having the lowest volume (Figure 1 and Table 2).

**TABLE 2:** Imaging biomarkers by diagnosis group.

Imaging biomarkers	CU pTau-	CU pTau +	CI pTau-	CI pTau +	*p*-value	Statistic
Amyloid-PET SUVR *n* = 51	12	7	7	25		
Frontal	0.992 (0.10)	1.318 (0.10)	1.013 (0.09)	1.468 (0.23)	< 0.001^°,*,∂,×^	*F* = 23.51
Post cingulate	1.158 (0.11)	1.515 (0.14)	1.196 (0.08)	1.684 (0.27)	< 0.001°^,*,∂,×^	*F* = 22.735
Lateral parietal	1.026 (0.10)	1.348 (0.13)	1.134 (0.18)	1.538 (0.25)	< 0.001^°,*,×^	*F* = 19.18
Temporal	0.972 (0.06)	1.254 (0.11)	1.034 (0.05)	1.391 (0.23)	< 0.001^°,*,×^	*F* = 17.757
Centiloid	12.994 (13.85)	65.844 (18.81)	22.324 (11.82)	90.08 (32.89)	< 0.001^°,*,∂,×^	*F* = 29.107
FDG-PET SUVR *n* = 67	26	17	6	18		
Whole gray matter	1.578 (0.074)	1.519 (0.078)	1.560 (0.13)	1.500 (0.10)	0.1	*F* = 2.178
Anterior cingulate cortex	1.504 (0.11)	1.428 (0.10)	1.478 (0.19)	1.425 (0.11)	0.318	*F* = 1.197
Precuneus	1.93 (0.13)	1.808 (0.13)	1.877 (0.20)	1.743 (0.17)	0.006*	*F* = 4.547
M1	1.704 (0.12)	1.615 (0.15)	1.710 (0.11)	1.620 (0.11)	0.114	*F* = 2.064
Superior frontal gyrus	1.646 (0.10)	1.571 (0.12)	1.668 (0.19)	1.57 (0.14)	0.264	*F* = 1.358
Superior parietal cortex	1.680 (0.14)	1.574 (0.12)	1.642 (0.14)	1.540 (0.14)	0.035*	*F* = 3.065
Posterior cingulate cortex	1.811 (0.11)	1.719 (0.13)	1.789 (0.21)	1.644 (0.17)	0.014*	*F* = 3.822
Parahippocampal gyrus	1.115 (0.06)	1.065 (0.07)	1.109 (0.09)	1.017 (0.09)	0.004*	*F* = 4.893
Hippocampus	1.282 (0.06)	1.241 (0.05)	1.258 (0.12)	1.173 (0.09)	< 0.001^*,∞^	*F* = 7.484
Amygdala	1.203 (0.06)	1.172 (0.07)	1.184 (0.11)	1.129 (0.10)	0.032*	*F* = 3.139
Middle frontal cortex	1.681 (0.10)	1.586 (0.12)	1.731 (0.22)	1.569 (0.12)	0.050^*,×^	*F* = 2.756
MPRAGE *n* = 259	102	36	39	82		
AD signature GM volume	0.113 (0.004)	0.113 (0.005)	0.111 (0.006)	0.107 (0.006)	< 0.001^*,∞,×^	*F* = 27.502
WMH (mm^3^)	4.474 (6.53)	6.494 (8.36)	7.537 (7.73)	7.341 (7.76)	0.135	*F* = 1.872
HOC	0.978 (0.01)	0.968 (0.02)	0.962 (0.03)	0.943 (0.04)	< 0.001^∙,*,∞,×^	*F* = 20.847
GMV (mm^3^)	594.39 (53.25)	594.677 (55.82)	587.264 (65.15)	575.036 (60.18)	< 0.001	*F* = 6.287
WMV (mm^3^)	463.269 (59.64)	466.24 (59.17)	471.892 (64.43)	457.796 (60.85)	0.164	*F* = 1.717

Demographic, neuropsychological, and MRI characteristics of the CU individuals from the Amyloid-PET, FDG-PET, and VBM and SBM analysis (PET analyses a subgroup of these individuals). Values are mean [SD (standard deviation)], ANOVA run with age and sex as covariates. GMV, gray matter volume; WMV, white matter volume; WMH, white matter hyperintensity volume; HOC, hippocampal occupancy score, composite score with no units, mm; millimeter. Symbols represent significant *post-hoc* tests from multiple comparisons Bonferroni tests between diagnosis and pTau groups: °CU pTau- vs. CU pTau + ; ^∙^CU pTau- vs. CI pTau-; *CU pTau- vs. CI pTau + ; ^∂^CU pTau + vs. CI pTau-; ^∞^CU pTau + vs. ^×^CI pTau + ; CI pTau- vs. CI pTau + .

**FIGURE 2:**
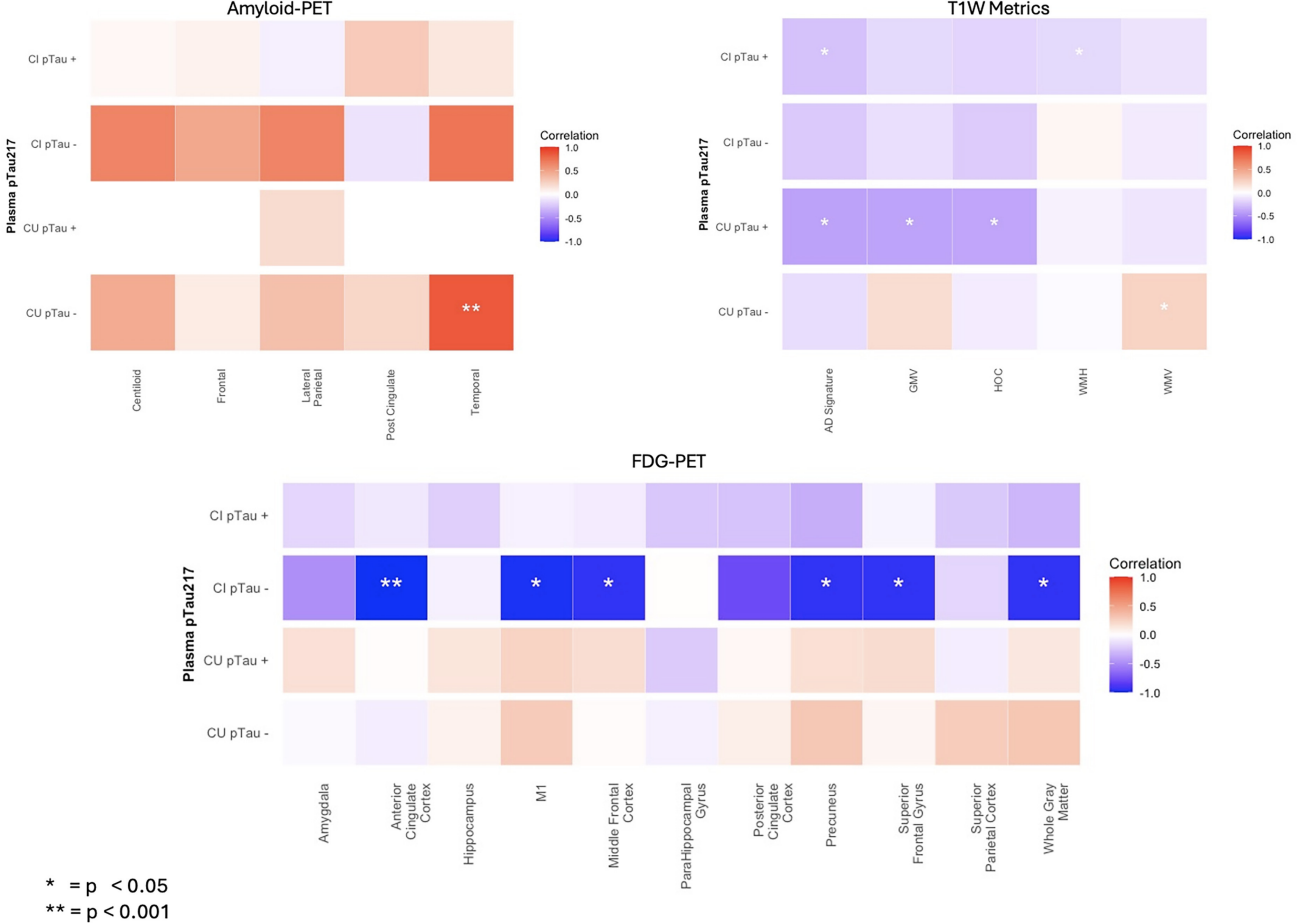
Correlation matrix between pTau-217 and brain imaging metrics across Amyloid-PET SUVR and centiloid, T1-weighted metrics, and FDG-PET SUVR. Correlation matrix showing Pearson r values between pTau-217 and selected biomarkers, stratified by diagnostic group, covariates included age and sex. Significance represented by *. *P*-value subjected to false discovery rate using Benjamini and Hochberg method. *p < 0.05, **p < 0.01.

Spearman correlation analyses (two-tailed) examining the regional relationships between plasma p-Tau217 and Amyloid-PET lobe-based SUVR and Centiloid values (Table 3) showed that in the CU pTau- group, the temporal SUVR had a strong positive significant relationship to p-Tau217, no other group had any significant relationships, with the CU pTau + group not having enough power for correlation analysis given the small subgroup with Amyloid-PET data. In our analysis of p-Tau217 and FDG-PET regional hypometabolism, we found that in CI pTau - individuals, higher p-Tau217 was significantly correlated to whole gray matter, anterior cingulate cortex, precuneus, M1, superior frontal gyrus and middle frontal cortex hypometabolism. No other groups had significant relationships of p-Tau217 and FDG-PET. In our analysis of p-Tau217 and MPRAGE derived metrics in the CU pTau- group, we found a positive relationship with p-Tau217 and White Matter Volume. CU pTau + individuals had a significant negative relationship between p-Tau217 and gray matter volume in the AD Signature regions, HOC and overall gray matter volume. CI pTau + had a negative significant relationship between p-Tau217 and gray matter volume in the AD Signature regions and WMH volume. Correlation plots for all data from Table 3 are shown in Figure 2.

**TABLE 3:** Correlations between plasma p-Tau217 and MRI, amyloid-PET and FDG-PET metrics.

p-Tau217	CU pTau-	CU pTau +	CI pTau-	CI pTau +
MPRAGE (n = 243)				
AD signature GM volume	−0.147	−0.408*	−0.239	−0.269*
WMH (mm^3^)	−0.021	−0.055	0.048	−0.229*
HOC	−0.085	**−0.392**	−0.226	−0.188
GMV (mm^3^)	0.187	−0.4*	−0.139	−0.167
WMV (mm^3^)	0.237*	−0.107	−0.091	−0.12
Amyloid-PET SUVR (n = 35)				
Frontal	0.107	.	0.468	0.071
Post cingulate	0.22	.	−0.119	0.286
Lateral parietal	0.342	0.188	0.651	−0.068
Temporal	0.863**	.	0.726	0.129
Centiloid	0.455	.	0.651	0.036
FDG-PET SUVR (n = 51)				
Whole gray matter	0.304	0.126	−0.961*	−0.313
Anterior cingulate cortex	−0.078	0.016	−1*	−0.1
Precuneus	0.301	0.17	−0.961*	−0.358
M1	0.282	0.239	−0.989*	−0.061
Superior frontal gyrus	0.05	0.191	−0.961*	−0.046
Superior parietal cortex	0.277	−0.076	−0.177	−0.235
Posterior cingulate cortex	0.089	0.035	−0.789	−0.262
ParaHippocampal gyrus	−0.064	−0.229	0.01	−0.246
Hippocampus	0.071	0.14	−0.062	−0.211
Amygdala	−0.024	0.167	−0.492	−0.177
Middle frontal cortex	0.023	0.181	−0.961*	−0.086

Table represents data from the Spearman’s correlations using age and sex as covariates within pTau positive and negative groups. GMV, gray matter volume; WMV, white matter volume; WMH, white matter hyperintensity volume; HOC, hippocampal occupancy score; composite score with no units, mm; millimeter. *p < = 0.05, **p < 0.01. Bold values indicate statistically significant correlations after false discovery rate correction.

In the voxel-based morphometry analysis of the regression of p-Tau217 and gray matter volume across all diagnosis and p-Tau217 groups, higher p-Tau217 was significantly associated with decreased volume in the limbic (hippocampus), inferior and superior temporal cortices, angular gyrus, bilateral superior frontal gyrus, supramarginal gyrus, precuneus and left inferior occipital gyrus (*p* < 0.05 FWE corrected, Table 4). In the surface-based analysis of p-Tau217 and cortical thickness across all diagnosis groups, higher p-Tau217 was significantly associated with decreased thickness across the limbic (parahippocampaus), fusiform gyrus, left inferior temporal gyrus, left middle and inferior frontal gyrus, the precuneus and left inferior parietal gyrus, left precentral, left cingulum, and left posterior cingulate gyrus (*p* < 0.05 FWE corrected, Table 4). There was a significant relationship between higher p-Tau217 and a higher gyrification index in the left middle frontal gyrus, and higher fractal dimension in the left anterior cingulum across all groups (*p* < 0.05 FWE corrected, Table 4).

**TABLE 4:** Significant results from morphometric analysis of p-Tau217 in all individuals.

All group analysis *N* = 259	Cluster size (vertex/voxel)	FWE peak *p*-value	*T*	*Z*	Coordinates	Brain region
					**(mm mm mm)**	
Gray matter volume (VBM)						
	25,080	< 0.001	7.1	6.78	−27 −10 −24	L Hippocampus
	20,317	< 0.001	6.66	6.39	50 −21 −20	R Inferior Temporal gyrus
	923	< 0.001	5.69	5.52	58 −66 28	R Angular
	566	< 0.001	5.67	5.49	20 8 54	R Superior Frontal gyrus
	724	0.002	5.24	5.1	−42 10 12	L Insula
	100	0.006	4.94	4.83	−34 −76 −10	L Inferior Occipital gyrus
	90	0.01	4.81	4.7	−30 3 50	L Middle Frontal gyrus
	227	0.011	4.79	4.69	3 8 2	R Caudate
	148	0.017	4.69	4.59	50 14 −24	R Superior temporal gyrus
	32	0.03	4.54	4.45	−22 22 56	L Superior Frontal gyrus
	57	0.032	4.52	4.43	66 −44 28	R Supramarginal gyrus
	24	0.036	4.49	4.4	39 −86 36	R Precuneus
Thickness (SBM)						
	3,631	< 0.001	6.26	6.04	−53 −30 −22	L Inferior Temporal gyrus
	2,112	< 0.001	6.16	5.94	44 −26 −23	R Fusiform gyrus
	470	< 0.001	5.55	5.38	−8 −48 31	L Posterior Cingulate gyrus
	382	< 0.001	5.4	5.25	−27 17 40	L Middle Frontal
	260	< 0.001	5.28	5.14	−51 −51 45	L Inferior Parietal Lobule
	255	0.001	5.07	4.95	9 −51 28	R Precuneus
	180	0.001	5.07	4.94	−8 15 33	L Cingulum
	138	0.006	4.59	4.49	−38 1 29	L Precentral
	67	0.011	4.44	4.35	−41 27 24	L Inferior Frontal
	119	0.017	4.33	4.25	27 −31 −21	L Parahippocampus
	89	0.017	4.33	4.25	−32 −60 39	L Inferior Parietal gyrus
	14	0.03	4.17	4.1	18 −69 33	L Precuneus
	25	0.033	4.15	4.08	34 −24 8	R Claustrum
	13	0.036	4.12	4.05	46 −74 16	R Middle Occipital gyrus
*Fractal dimension (SBM)						
	80	0.01	4.52	4.43	−4 34 −1	L Anterior Cingulum
	10	0.028	4.25	4.18	10 38 24	R Anterior Cingulum
*Gyrification index (SBM)						
	35	0.034	4.09	4.02	−36 55 −9	L Middle Frontal gyrus
Sulcal depth (SBM)						
	44	0.028	4.13	4.06	−4 14 25	L Anterior Cingulum

Results are listed at a threshold of *p* < 0.05 FWE corrected, primary peaks within cluster listed in table. Coordinates listed are Montreal Neurological Institute. All results are from the regression of pTau-217 plasma values across voxel and surface imaging values in the negative direction except those results listed with a *, which were in the positive direction. L, Left; R, Right.

**FIGURE 3:**
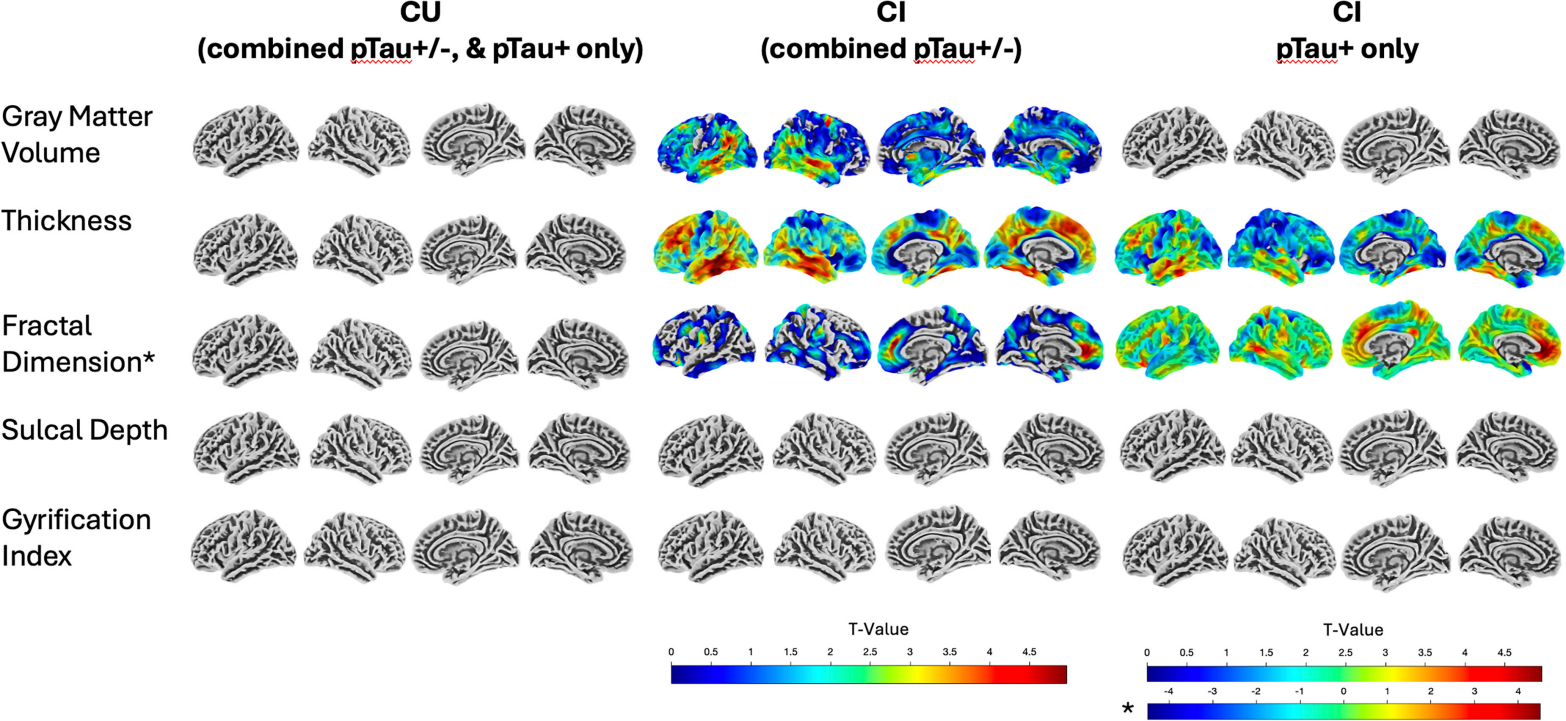
Relationship of p-Tau217 with brain morphometry. Surface overlays show significant results from diagnosis and pTau ± stratified analyses of p-Tau217 from voxel based morphometry (gray matter volume) analysis and surface-based morphometry (SD, FD, CT, and GI) covarying for age, sex, and education. *FD analysis revealed both a positive and negative relationship between FD and p-Tau217, all other analyses were a negative relationship between brain morphometry and p-Tau217. The color bar represents *t*-value.

When stratifying the VBM and SBM analysis between CU and CI groups, we found that CU did not have any significant relationships between p-Tau217 and regional gray matter volume, cortical thickness, FD, SD, or GI, either as a whole group or also when looking at the pTau + individuals alone. In the voxel-based regression of p-Tau217 and gray matter volume within the CI group, higher p-Tau217 was significantly associated with decreased gray matter volume in the left middle temporal gyrus (*p* = 0.003 FWE corrected), and the right anterior cingulate (*p* = 0.05 FWE corrected). In the SBM analysis, we found that higher p-Tau217 was significantly associated with decreased thickness in the left inferior temporal gyrus, left middle frontal gyrus, left middle temporal gyrus, right fusiform gyrus, right superior temporal gyrus, and left inferior temporal gyrus (*p* < 0.05 FWE corrected). There was a significant relationship between higher p-Tau217 and a higher fractal dimension in the bilateral anterior cingulate in CI individuals, as well as when looking only at CI PTau + individuals alone (*p* < 0.05 FWE corrected). All results are shown in Table 5 and displayed in Figure 3.

**TABLE 5:** Significant results from morphometric analysis of p-Tau217 in CI, and CI p-Tau positive only individuals.

CI, CI p-Tau +	Cluster size (vertex/voxel)	FWE peak *p*-value	T	Z	Coordinates	Brain region
Gray matter (VBM)						
CI whole group	11,352	0.003	5.3	5.01	−54 −51 24	L Middle temporal gyrus
	1,047	0.02	4.79	4.56	21 6 52	R Superior Frontal gyrus
Thickness (SBM)						
CI whole group	81	0.006	4.69	4.48	−53 −33 −20	L Inferior temporal gyrus
	68	0.007	4.63	4.43	−26 16 41	L Middle frontal gyrus
	177	0.009	4.57	4.37	−50 −40 −3	L Middle temporal gyrus
	62	0.014	4.47	4.28	40 −38 −24	R Fusiform gyrus
	26	0.024	4.3	4.14	48 −45 9	R Superior temporal gyrus
	20	0.032	4.22	4.06	−43 −51 −13	L Inferior temporal gyrus
CI pTau-217 + only	1,234	0.030	4.34	4.1	−36 −44 −22	L Fusiform gyrus
Fractional dimension (SBM)						
CI whole group*	255	0.045	4.23	4.07	−7 42 5	L Anterior cingulate
	88	0.05	4.2	4.04	12 42 19	R Anterior cingulate
CI pTau-217 + only	230	0.032	4.43	4.17	19 −79 19	R Cuneus
CI pTau-217 + only*	516	0.008	4.84	4.51	−9 21 9	L Anterior cingulate

Results are listed at a threshold of *p* < 0.05 FWE corrected, primary peaks within cluster listed in table. Coordinates listed are Montreal Neurological Institute. All results are from the regression of pTau-217 plasma values across voxel and surface imaging values in the negative direction except those results listed with a *, which were in the positive direction. L, Left; R, Right.

## 4 Discussion

In this study, we sought to determine whether AD neuroimaging biomarkers are increased in individuals with elevated p-Tau217. This study also uniquely investigates the relationship of plasma p-Tau217 to both whole brain morphometric and regional imaging biomarkers across several modalities (PET, MRI) in CU and CI pTau + and pTau- individuals. Our findings demonstrate that plasma p-Tau217 is elevated in individuals with CI compared to CU adults, with CU pTau + individuals having some early AD neuroimaging biomarker signals in amyloid-PET and brain structure, aligning with its role as a scalable index of AD biology adults (Janelidze et al., 2024; Palmqvist et al., 2024; Ossenkoppele et al., 2025).

Individuals with elevated plasma p-Tau217 had increased amyloid-PET deposition across the cortex, as well as significantly higher centiloid scores, both in CU and CI individuals, underscoring the sensitivity of p-Tau217 to preclinical amyloid accumulation (Palmqvist et al., 2020; Janelidze et al., 2024). We saw in that p-Tau + CI individuals had higher amyloid-PET Aβ deposition across all regions, and CU p-Tau + individuals had higher amyloid-PET Aβ compared to CU p-Tau- individuals as well. This is consistent with recent studies showing p-Tau217’s high accuracy in detecting amyloid positivity even in asymptomatic cohorts (Mattsson-Carlgren et al., 2020; Ossenkoppele et al., 2021), often outperforming other plasma markers like p-tau181 or Aβ42/40 ratios(Ashton et al., 2024). For instance, in a large population-based study, plasma p-Tau217 predicted amyloid-PET positivity with AUCs exceeding 0.90 in CU adults, highlighting its utility for early screening (Thijssen et al., 2021). Moreover, a longitudinal analysis in the Swedish BioFINDER study showed that amyloid-β-positive CU individuals had accelerated, increased p-Tau217 over time compared to amyloid-β-negative CU individuals, and p-Tau217 correlated with worsening of cognition and temporal lobe atrophy in CU individuals (Mattsson-Carlgren et al., 2020).

In our analysis, both CU and CI pTau + individuals had lower gray matter volume in the AD Signature region of interest encompassing limbic and temporal lobe regions as well as hippocampal occupancy score. Structurally, the correlation between p-Tau217 and HOC in CU pTau + individuals indicates p-Tau217’s link to hippocampal integrity, potentially capturing early (preclinical) atrophy or ventricular expansion in these unimpaired individuals, similar to other studies showing some signal with p-Tau217 in the hippocampus (Jagust, 2018; Mattsson-Carlgren et al., 2020; Ossenkoppele et al., 2021; Janelidze et al., 2024). Voxel-based morphometry (VBM) and surface-based morphometry (SBM) revealed associations between p-Tau217 and decreased gray matter volume and cortical thickness across limbic, temporal, parietal, and frontal regions across all groups. In CI, elevated p-Tau217 was associated with reduced voxel-wise gray matter volume and cortical thickness in limbic, temporal, parietal, and frontal regions, patterns mirroring Braak staging of tau spread from MTL to neocortex (Sanchez et al., 2021). Increased fractal dimension in cingulate may indicate adaptive complexity or pathology-induced folding changes, a novel finding warranting replication (Brickman et al., 2022). Our results are similar to one by Thijssen et al. in MCI individuals, which found levels of p-Tau217 negatively associated with temporo-parietal GM volumes in their voxel-wise analysis (Thijssen et al., 2021). Of the several other studies looking at plasma p-Tau217 volumetric measures, each focused on regions of interest as opposed to whole-brain vertex approaches, with Ossenkoppele et al. (2021), identifying negative correlations with p-Tau217 and AD signature cortical thickness and hippocampal volume, Mielke et al., with WMH, diffusion measures, and temporal lobe cortical thickness (Mielke et al., 2021). These suggest progressive neurodegeneration, with p-Tau217 tracking tau-related atrophy in temporolimbic networks (Bejanin et al., 2017; Ottoy et al., 2024).

However, the lack of significant p-Tau217 correlations across voxel and surface analyses in CU alone supports our hypothesis of subtle, non-significant morphometric changes at this stage, where pathology may not yet manifest as detectable volume loss at a global level given the number of corrections done (Ossenkoppele et al., 2024). These results support p-Tau217 as a minimally invasive biomarker that reflects limbic-predominant changes in the preclinical stages and extends to broader neocortical involvement in symptomatic disease.

In our analysis of FDG-PET regional hypometabolism between groups, there was a significant overall difference between the 4 groups in the precuneus, superior frontal gyrus, posterior cingulate cortex, parahippocampal gyrus, hippocampus, amygdala and middle frontal cortex, with *post-hoc* tests showing that this was particularly driven by the lower glucose metabolism across regions in the CI pTau + group compared to the CU pTau- group. This aligns with evidence that p-Tau217 reflects subtle hypometabolism in limbic areas possibly before tau-PET abnormalities emerge (Groot et al., 2023; Quispialaya et al., 2025).

CI pTau- individuals had correlations between p-Tau217 and FDG-PET hypometabolism variation in several regions across the cortex, likely due to biological heterogeneity of the small group, and should be validated in a larger sample. MCI cohorts often encompass diverse etiologies, including non-AD pathologies, variable progression rates, and mixed amyloid/tau profiles (Ferreira et al., 2020). This absence of associations echoes findings from meta-analyses indicating weaker biomarker concordance in MCI compared to CU or AD, attributed to transitional stages and subtype variability (Antonioni et al., 2025).

These multimodal associations reinforce p-Tau217’s alignment with ATN framework, outperforming other tau isoforms in reflecting amyloid, tau, and neurodegeneration (Moscoso et al., 2021; Ashton et al., 2022). Functionally, the intersection hypothesis posits that amyloid drives neuronal hyperactivation and blood-brain barrier leakage, elevating plasma p-Tau217 as a proxy for intersecting amyloid-tau-neurodegeneration processes. Our limbic-focused findings in CU support this, with early hippocampal hypometabolism in CU pTau + individuals, and decreased hippocampal occupancy potentially preceding other cortical neuroimaging biomarker signals (Berron et al., 2021). Compared to postmortem-validated imaging, our results echo MTL vulnerability, where p-Tau217 correlates with subregional tau patterns (Ravikumar et al., 2024).

This study has several limitations that should be considered when interpreting the findings. These limitations include the cross-sectional design, limiting causal inferences, thus future analysis on longitudinal follow-up could track p-Tau217 with progression. Another limitation of this study is that cardiovascular risk factors and use of anti-dementia medications were not systematically modeled, and future studies in larger samples should examine their potential influence on p-Tau217 and neuroimaging measures. Moreover, because of sample size considerations, we could not stratify analyses by APOE ε4 genotype despite differences in ε4 distributions between groups, and thus future studies should examine associations between the presence of the ε4 allele and its impact on p-Tau217 across the AD continuum. Further, although merging MCI and AD into a cognitively impaired group improved statistical power to investigate pTau elevated and non-elevated subgroups, some analyses, particularly those involving FDG-PET, remain limited by small sample sizes. Our cohort’s demographic (e.g., higher female proportion in CU) may also limit generalizability, however we controlled for sex in all analyses. Future studies should incorporate tau-PET for direct comparisons and diverse populations to assess racial/ethnic differences in p-Tau217-neuroimaging links (Mendes et al., 2024). Nevertheless, these results highlight the potential of plasma p-Tau217 for scalable risk stratification and underscore the need for larger, longitudinal studies to further validate these findings.

## 5 Conclusion

In conclusion, we identified increased amyloid-PET signal, hippocampal hypometabolism and volume change in cognitively impaired individuals with elevated pTau-217, as well as more widespread amyloid-PET, hypometabolic and brain structure changes associated with atrophy in cognitively impaired individuals with increased pTau-217. Thus, plasma p-Tau217 offers a promising, non-invasive tool for detecting early temporolimbic structural neuroimaging changes in AD, with strong concordance to amyloid-PET and MRI, and emerging utility in FDG-PET. These findings advocate for its integration in clinical triage, risk stratification, and trial enrichment, particularly in preclinical stages.

## Data Availability

The data analyzed in this study is subject to the following licenses/restrictions: Data can be obtained from the KU ADRC upon request on our website. Requests to access these datasets should be directed to https://redcap.kumc.edu/surveys/?s=M749NL4AAEPJAAT4.

## Footnotes

1
http://dbm.neuro.uni-jena.de/cat/

2
http://www.fil.ion.ucl.ac.uk/spm/software/spm12/

3
http://neuromorphometrics.com/

4
http://www.neuro.uni-jena.de/cat12/CAT12-Manual.pdf
